# Subacute Endocarditis in Children

**Published:** 1917-07-28

**Authors:** C. Willett Cunnington


### SUBACUTE ENDOCARDITIS IN CHILDREN.

By C. WILLETT CUNNINGTON, M.B., B.C.

THIS is a disease of common occurrence in certain neighbourhoods, especially those situated in clay soil and exposed to damp. It is most prevalent not, as might be supposed, in winter, but in summer. That it is a disease only too frequently overlooked is proved by the fact that a large number of adults with well-established valvular defects can give no history of illness to explain the source of their heart lesions; many have never had "rheumatic fever" or scarlet fever; in these the valvular trouble must have arisen in some more subtle fashion. Probably they suffered in youth from unrecognised subacute endocarditis. Indeed, it is no rarity to find children actually suffering from this disease who have been recently under medical care and passed as having nothing important.

That such regrettable oversights are made is due, probably, to the notion that endocarditis is an *acute* disease with pronounced physical signs. The existence of the subacute forms is often forgotten. It lurks as a pitfall for the unwary. It occurs in children, especially those with fair complexions, between the fifth year and puberty. There is often a history of "growing pains," and its definite onset is frequently preceded by a succession of vague "feverish attacks," which have been ascribed to "influenza" (always a hazardous diagnosis to make in young children). The child's health gradually becomes impaired, headaches and lassitude are noticed, but there are as yet none of the orthodox cardiac symptoms. A cursory examination reveals nothing. Later, after weeks, or even months, of persistent ill-health, a more thorough examination shows a gravely damaged endocardium. The presence of subacute endocarditis should have been discovered in the earlier stages, for it is only at that period that there is much hope of obtaining a cured heart with no trace of damage to the valves.

Of cardinal importance is a careful and prolonged series of observations of the temperature. It is strange how such a simple instrument as the thermometer is forgotten as a means of diagnosis. But it must be employed with proper precautions. When slight fever is suspected it is useless to employ even the most sensitive thermometer for the bare two or three minutes commonly considered sufficient. It should be left in the mouth for at least *seven* minutes, and readings should be taken three times a day, one of which should be about 5 P.M. In subacute endocarditis a daily rise to 99° or 99.5° is the rule.

A persistent slight fever in a child is highly suggestive of this disease, provided that other causes of fever without gross physical signs can be excluded. These other causes are typhoid fever, tuberculosis, pyorrhœa, infections of the urinary tract, and obstinate constipation. Having excluded these, attention should be concentrated on the heart whenever a child's temperature is found to be raised for over a week without physical signs.

The child is kept in bed and the cardiac dulness carefully watched for signs of slight but increasing dilatation.

This, combined with irregularity of the pulse, suggests active myocarditis. The pulse may be either intermittent or irregular in rhythm; the latter is the commoner. In the first week or so there may be no dilatation recognised, and the sounds may seem clear. Eventually one or more murmurs develop. A diastolic murmur is conclusive of endocarditis. A systolic murmur, which may be only a blurring of the first sound, developing presently into a definite soft blowing murmur, may have the clinical appearance of a "functional murmur." But taken in conjunction with the persistent fever it is to be regarded as almost certainly organic, in the sense that the endocardium is actively inflamed. How far the inflammation affects the myocardium, and how far the endocardium, is in practice of little importance, for the treatment of both is similar. The longer the fever persists, the more certain becomes the diagnosis.

There are, then, three stages before the diagnosis is established. First, persistent fever without physical signs; second, the appearance of cardiac signs of uncertain nature; third, the development of conclusive signs of endocarditis. An observer may see the patient for the first time in the second or third stage, when his suspicions should be at once excited. His task is more difficult should he see the case at the beginning of the first stage.

The child does not seem ill, there is an initial temperature of 100° to 102°, and examination shows nothing but some redness of the fauces. A throat swab for diphtheria having proved negative, a diagnosis of simple slight tonsillitis is made. But it is wise to regard such an attack as being of a rheumatic nature, and to assume that it is capable of further rheumatic developments. If the temperature does not fall in five or six days, the closest watch on the heart is necessary.

If the case is first seen in the second stage, that is to say when an apparently functional murmur is heard, the temperature should be examined for a week before the murmur is classed as being only "hæmic" in nature.

It is to be remembered that a recent organic murmur does not obey the rules of old-established organic murmurs. It is not necessarily traceable into the axilla; it is apt to be very soft, but it is more audible in the erect than in the horizontal posture. Lastly, in subacute endocarditis the blood shows a slight leucocytosis.

That the disease is an infection is almost certain, but the nature of the germ is doubtful. It is probably a streptococcus, and many regard it as being derived from some part of the alimentary tract. Its point of entry is usually the tonsils, but it may occasionally be the appendix.

The prognosis is speculative; from our experience of adults with old valvular lesions we can asssume that in many cases the infection must die out unrecognised, leaving a more or less damaged valve as its only trace. In others the adolescent form, after repeated exacerbations, develops into the classical "rheumatic fever."

Just as phthisis in the adult is often only a mature development of adolescent tuberculosis of the mediastinal glands, so, for aught we know, may rheumatic fever be a late stage of an earlier infection. It is certain that subacute endocarditis is very prone to relapse, and these relapses may at last run into one another so that a condition indistinguishable from "malignant endocarditis" may ensue.

The prognosis of subacute endocarditis, then, must necessarily be very guarded. An apparently slight attack may be the starting-point of a series culminating in a generalised blood infection; or it may leave the patient with a damaged valve and a marked liability to further mischief. A second attack will seldom leave a heart unscathed, and we cannot foretell whether the injury will be slight or severe. The treatment resolves itself into three stages; first, the waiting period when the diagnosis is uncertain. In this rest in bed is needed as a precautionary measure, and as a means of allowing closer observation to be made.

In the second stage, when the diagnosis is made, the rest must be of an absolute nature. The patient is to be treated as though it were a case of typhoid fever, and all physical movements forbidden. It is wise to allow no pillow and to raise the foot of the bed so as to secure a horizontal posture for the upper part of the body. The diet is strictly "rheumatic" in its limitations. Salicylates are usually given, but they seldom produce the marked effects in reducing the temperature such as one obtains in acute rheumatic fever. An icebag worn continuously over the heart for a week or so is probably more effective in lowering the temperature and in quieting the pulse.

On the assumption that the infection is a streptococcus derived from the alimentary tract, a double autogenous vaccine of *S. salivarius* and *S. fæcalis* is worthy of trial. Blood cultures are usually sterile, probably because the organisms in the sample of blood are scanty, and unable to grow in the presence of the anti-bodies contained in it; so that a direct means of obtaining the causal germ is at present unavailable.

In intractable cases two or three doses of normal horse serum given hypodermically may induce a change for the better. An aperient is generally necessary, especially as constipation is an aggravating factor in the disease. Liquid paraffin is preferable for this purpose, on the ground that it may possess some power of checking toxic absorptions from the intestine.

The duration of absolute rest depends on the length of time the fever lasts and the degree of cardiac dilatation left at the end of it.

The temperature seldom falls in less than a fortnight, and it may remain raised for months. A month to six weeks is a common time. The febrile stage being over, a long rest is required by the heart before the horizontal position can be modified. Two months is none too long.

One should wait until any dilatation has disappeared in the hope of securing a complete restoration to the normal. Nevertheless, in obstinate cases a certain amount of dilatation may persist, and if after prolonged rest this fails to improve further a continuation of absolute rest is probably useless. The heart may indeed be too flabby to retract, and may require cautious exercise to strengthen the myocardium. The patient is raised in bed by gradual stages for this purpose.

The greatest judgment is needed to discriminate between cases which are capable of deriving further benefit from rest and those where a certain amount of permanent enlargement is inevitable and in which rest can no longer produce improvement. Similarly, there are cases where the temperature persists indefinitely, and these may show an improvement in this respect only after a change of posture. The murmur is not in itself a guide, for the observer cannot tell whether it is going to remain permanently or whether it is due only to temporary dilatation.

Most mistakes are made in the direction of allowing too short a rest, an error of judgment encouraged by the natural desires of the parents; yet the other extreme is a pitfall to be avoided; *trop de zèle* in keeping a child lying flat for month after month may induce a hopelessly flabby cardiac muscle. Two months after the temperature has been normal the time arrives for experimenting with a change of posture. It is during this stage of increasing exercise and later, when walking is begun, that close watch for signs of relapse is needed. The thermometer is the best guide. It is wise to give salicylates from time to time throughout the next year.

A complication of subacute endocarditis is chorea. If we regard chorea as being essentially a group of symptoms caused by the rheumatic toxin acting upon a sensitive cerebral cortex, we shall not be surprised to find varying degrees of it appearing in our cases: some in which the chorea is a minor feature, others in which it is the most prominent. In the latter the treatment is modified by keeping the child in a darkened room and exhibiting arsenic. In children with subacute endocarditis chorea is frequently, perhaps generally, lurking in the background as it were, and easily brought forward by exciting influences.

Lastly, the presence of subcutaneous nodules, always indicative of endocarditis, though less common in the subacute type of the disease, is to be looked for.

